# Potential of *Aspergillus oryzae* as a biosynthetic platform for indigoidine, a non-ribosomal peptide pigment with antioxidant activity

**DOI:** 10.1371/journal.pone.0270359

**Published:** 2022-06-23

**Authors:** Sarocha Panchanawaporn, Chanikul Chutrakul, Sukanya Jeennor, Jutamas Anantayanon, Nakul Rattanaphan, Kobkul Laoteng

**Affiliations:** Functional Ingredients and Food Innovation Research Group (IFIG), National Center for Genetic Engineering and Biotechnology (BIOTEC), National Science and Technology Development Agency (NSTDA), Khlong Luang, Pathum Thani, Thailand; USDA Forest Service, UNITED STATES

## Abstract

The growing demand for natural pigments in the industrial sector is a significant driving force in the development of production processes. The production of natural blue pigments, which have wide industrial applications, using microbial systems has been gaining significant attention. In this study, we used *Aspergillus oryzae* as a platform cell factory to produce the blue pigment indigoidine (InK), by genetic manipulation of its non-ribosomal peptide synthetase system to overexpress the indigoidine synthetase gene (*AoinK*). Phenotypic analysis showed that InK production from the engineered strain was growth associated, owing to the constitutive control of gene expression. Furthermore, the initial pH, temperature, and glutamine and MgSO_4_ concentrations were key factors affecting InK production by the engineered strain. The pigment secretion was enhanced by addition of 1% Tween 80 solution to the culture medium. The maximum titer of total InK was 1409.22 ± 95.33 mg/L, and the maximum productivity was 265.09 ± 14.74 mg/L·d. Moreover, the recombinant InK produced by the engineered strain exhibited antioxidant activity. These results indicate that *A*. *oryzae* has the potential to be used as a fungal platform for overproduction of extracellular non-ribosomal peptide pigments.

## Introduction

Pigments are widely used in food, cosmetic, and textile industries to produce diverse colored products. The colorant market has been continually expanding worldwide [1–3]. However, because of concerns of the impact of synthetic colorants on health and environment, some synthetic colorants are banned. Evidence indicated that these synthetic colorants caused hyper allergenicity, carcinogenicity, and other toxicological problems [4, 5]. Plants and microorganisms are the main sources of natural pigments. However, the production of plant pigments has several drawbacks, such as land-use requirements, plant diseases, weather-dependent yields, and post-harvest processing. Microbial strains capable of biosynthesis of pigments provide a valuable alternative and are readily accessible for commercial production. Simple and feasible fermentation and downstream processes for microbial pigment production offer advantages over traditional pigment production from plant sources [1, 6, 7]. Red, orange, and yellow are common pigment shades produced by bacteria, yeast, and microalgae [8]. However, there is a high demand for other colors of spectra, such as blue shades, becuase of the scarcity of stable natural blue pigments in the market [9].

Indigoidine [5,5’-diamino-4,4’-dihydroxy-3,3’-diazadiphenoguinone-(2,2’)] is a water-insoluble dark blue pigment belonging to the pyridine class, which consists of two L-glutamine residues. However, the intrinsic mechanisms involved in the production of indigoidine remain elusive. Further research is required to elucidate the potential commercial applications of indigoidine. Indigoidine acts as both an antimicrobial substance and cryoprotectant [10, 11]. Based on the two-pyridone ring structure with carbonyl and amino groups, it has been postulated that the pigment possesses antiradical and antioxidant activities [12, 13]. Indigoidine is naturally produced by several groups of bacteria during the non-growth phase of cultivation [10, 13–17].

Indigoidine synthetase (INDS), a member of the non-ribosomal peptide synthetase (NRPS) family, biosynthesizes indigoidine by catalyzing the thioesterification of the two L-glutamines. In fact, the post-translational modification process is required for the conversion of inactive *apo-*form of INDS to the active *holo-*form. This evidence is accessed by introducing a coenzyme A-derived moiety into the thiolation (T)-domain of the enzyme with actions of a co-factor and 4’-phosphopantetheinyl transferase (PPTase) activity [17, 18]. When the INDS is become active, the biosynthesis of indigoidine is initiated by cyclization of a single molecule of glutamine through an intramolecular amide bond. The amino acid intermediate is then dehydrogenated, most likely by oxidation. Finally, the two modified cyclic glutamines condense to form the pigment [13, 19]. However, the pigment production yield from exotic microbial strains is comparatively low, which is impractical for industrial production. Genetic recombination improved the rate of indigoidine production in diverse microbes such as *Escherichia coli*, *Corynebacterium glutamicum*, and yeasts [16, 18, 20–22]. Among these genetic modified strains, a high titer of indigoidine was acquired by the overexpression of INDS and other relevant genes in conjunction with optimized fermentation conditions. However, some host systems may not be suitable for producing dipeptide pigments, because of the absence of a complex NRPS system. Aditionally, most pigments are intracellular metabolite products that require downstream processing, including cell disruption and extraction processes.

*Aspergillus oryzae* is a filamentous fungus that is generally recognized as safe (GRAS) [23]. *A*. *oryzae* has an amino acid precursor pool and a complete NRPS system; therefore, this fungus has been successfully used to produce functional peptides, such as β-lactam antibiotics [24]. Similar to other eukaryotes, this filamentous fungus is the host of choice for improving metabolite biosynthesis by self-tolerance mechanisms. These mechanisms include the transport of specific compounds to storage organelles such as vacuoles, modification of metabolites to generate less toxic forms, and cellular release of metabolites [25, 26]. In addition, studies have been conducted on the effects of nonionic surfactants, such as polyethylene glycol (PEG), Triton X, Tween, and Tergitol, on cell membrane structure, particularly on membrane integrity and permeability. These studies led to the development of the process for production of extracellular metabolites from filamentous fungi [27–31]. Recently, a fungal platform for oligopeptide production from *A*. *oryzae* has been established, by genetic manipulation of the endogenous NRPS system [32]. However, pigment production by using an NRPS-assisted mechanism in filamentous fungi has not yet been established.

Using a gene-editing approach, indigoidine production using *A*. *oryzae* was investigated in this study. The engineered strain of *A*. *oryzae* was constructed by overexpressing the gene encoding INDS, and the pigment production yield was evaluated under different variables in fungal fermentation. Furthermore, the pigment permeability with regard to extracellular production and cell morphology was investigated. This study explored the non-ribosomal peptide (NRP) biopigment overproduction by a filamentous fungal system through synthetic biology and physiological manipulation.

## Materials and methods

### Microbial strains and cultivations

The *pyrG-*deficient strain *A*. *oryzae* BCC 7051 was used as the recipient in this study [32]. The fungal strain was maintained on Czapek Dox medium supplemented with 0.5% (w/v) uridine and 0.2% (w/v) uracil. Spore inoculum was prepared by cultivating the fungal cells on rice medium at 30°C for 5–7 d; 0.05% (v/v) Tween 80 solution was used to harvest the spores. Spore suspension at a final concentration of 2 × 10^6^ spores/mL was inoculated into a 250-mL Erlenmeyer flask. The flask contained 50 mL of a semi-synthetic medium (SM), consisting of 4.0% (w/v) glucose, 0.5% (w/v) yeast extract, 0.02% (w/v) NH_4_Cl, 0.24% (w/v) KH_2_PO_4_, 0.05% (w/v) MgSO_4_ · 7H_2_O, 0.01% (w/v) CaCl_2_ · 2H_2_O, 0.0015% (w/v) FeCl_3_ · 7H_2_O, 0.001% (w/v) MnSO_4_ · H_2_O, and 0.008% (w/v) ZnSO_4_ · 7H_2_O) [33]. Cultures were incubated at 30°C in a rotary shaker at 200 rpm. Mycelial cells were harvested at different time points to determine biomass, residual glucose, and blue pigment concentrations.

*E*. *coli* DH5α (*supE44*, *ΔlacU169*, (*Φ80*, *lacZΔM15*), *hsdR17*, *recA1*, *endA1*, *gyrA96*, *thi1*, *relA1*) (Thermo Fisher Scientific, USA) was used as the recipient for propagation of the constructed recombinant plasmids. *E*. *coli* transformant was grown in Luria–Bertani (LB) medium containing 100 mg/L ampicillin at 37°C with shaking at 200 rpm.

*Saccharomyces cerevisiae* strain INVSCI (*MAT*α, *his3-Δ1*, *leu2*, *trp1-289*, *ura3-52*, *MAT*, *his3-Δ1*, *leu2*, *trp1-289*, *ura3-52*) (Invitrogen, USA) was used for plasmid construction. The yeast was grown in either a complete medium, YPD (consisting of 1.0% (w/v) bacto-yeast extract, 2.0% (w/v) bacto-peptone, and 2.0% (w/v) glucose), or a selective medium, SD (consisting of 0.67% (w/v) bacto-yeast nitrogen base and 2.0% (w/v) glucose). Both media were supplemented with L-tryptophan (20 mg/L), L-histidine hydrochloride (20 mg/L), and L-leucine (30 mg/L). Yeast cultures were incubated at 30°C with shaking at 200 rpm.

### Construction of the indigoidine-producing strain of *A*. *oryzae* (*AoInK*)

The 4134-bp DNA sequence of *A*. *oryzae* (*AoinK*) was designed from the coding region of the *ScindC* gene of *Streptomyces chromofuscu*s (S1 Table). The design was based on the general rule of RNA stability and the codon usage of *A*. *oryzae* using the OptimumGene^™^ algorithm by Genscript (Piscataway, USA). We synthesized the *AoinK* fragment using Genscript and then subcloned it into the pUC57 plasmid. DNA fragments, including the *gpdA* promoter of *A*. *nidulans* (*AnPgpdA*), *AoinK*, *nos3* terminator of *Agrobacterium tumefaciens*, and *AfpyrG* marker, were prepared by PCR, using Platinum^™^Taq DNA Hi Fidelity polymerase (Invitrogen, USA). Using 20-bp overlapping primer sets (S2 Table), 4 DNA fragments and EcoRI-linearized pPNGB plasmid [34] were co-transformed into *S*. *cerevisiae* cells using the PEG/lithium acetate method [35]. The resulting plasmid, pAoInK-pyrG, was extracted from the yeast transformant using Zymoprep^™^ Yeast Plasmid Miniprep II (Zymo Research, USA) and then shuttled into *E*. *coli* for propagation. Restriction enzyme analysis and DNA sequencing were performed to verify the accuracy of plasmid construction. The construction scheme for the pAoInK-pyrG plasmid is shown in S1 Fig.

The constructed plasmid (1–3 μg) was transformed into the *pyrG-*deficient strain of *A*. *oryzae* using the PEG-mediated method (PMT) [36–38] with a modification to the enzyme cocktail concentration. Fungal protoplast cells were prepared and suspended in ice-cold sorbitol / Tris–HCl / CaCl_2_ solution until they were used for DNA transformation. The transformed protoplasts were regenerated in potato dextrose broth with 1.2 M sorbitol for 2 h. The protoplasts were then immediately plated on Czapek Dox medium without nutrient supplementation. After incubation at 30°C for 10–14 d, the transformant colonies were selected based on the dark blue phenotype. Spore reisolation was conducted to obtain a pure colony of the AoInK strain.

### Submerged fermentation of AoInK strain

For submerged fermentation, we used SM as the basal medium and performed fungal cultivation for 5 d. Initial pHs (4.5, 5.5, 6.5, and 7.5) were the variables for indigoidine production by the AoInK strain. Furthermore, the effects of culture temperatures (25°C, 30°C, and 35°C) were further investigated by using the optimal initial pH value. Subsequently, under the optimal conditions of temperature and initial pH, we further investigated the effects of L-glutamine concentrations (2, 5, and 10 mM) and MgSO_4_ concentrations (2, 5, 10, and 50 mM), for optimization of indigoidine production by the AoInK strain.

### Effect of nonionic surfactants on extracellular pigment production

The effects of polyethylene glycol-4000 (PEG-4000), Tergitol NP-40, TritonX-100, and Tween 80 (Sigma-Aldrich, USA) at different concentrations (1% (w/v), 3% (w/v), and 5% (w/v)) were investigated. Each preparation was individually added to the culture medium. Fungal cultures were grown at 25°C for 5 d with shaking at 200 rpm. The following fermentation parameters were assessed: biomass yield (g/L), InK yield on glucose (Y_p/S_, mg/g), InK yield on biomass (Y_p/x_, mg/g), and InK productivity (mg/L·d).

### Determination of biomass titer and residual glucose concentration

The culture samples were filtered to separate the mycelial cells and fermented broth through Miracloth (Merck Millipore, Germany). Mycelial cells were hot-air-dried until their weight was constant. The residual glucose concentration in the fermented broth was measured using a high-performance liquid chromatography (HPLC) column (Ultimate 3000, Thermo, USA) equipped with a refractive index detector and an Aminex^®^ HPX-87H ion exclusion column (300 × 7.8 mm, 9-μm particle size, Bio-Rad Laboratories, USA), under isocratic mode with 18 mM H_2_SO_4_ solution as the mobile phase at a flow rate of 0.6 mL/min at 60°C for 30 min. Residual glucose concentration was calculated using a calibration curve of glucose standard at concentrations of 0.1–10.0 mg/mL.

### Extraction and quantification of indigoidine

Fresh fungal mycelia (50 mg) were ground with liquid nitrogen and then subjected to extraction of indigoidine by adding 1 mL of dimethyl sulfoxide (DMSO) with sonication, as previously described [18]. For the extracellular indigoidine extraction, the cell-free broth was diluted with 100% (v/v) DMSO solution. The prepared samples were subjected to quantification of indigoidine using a spectrophotometer (Jasco V-730BIO) at a wavelength of 612 nm. A calibration curve for calculating pigment concentration was generated using an indigoidine standard with known concentrations (ViabLife, PRC).

To analyze the indigoidine pigment, 10 μL of the sample dissolved in DMSO was subjected to reversed-phase high-performance liquid chromatography (RP-HPLC) column (Ultimate 3000, Thermo, USA) equipped with a diode-array detector (DAD) and C-18 Acclaim^™^120 column (4.6× 250 mm, 5-mm particle size, Bio-Rad Laboratories, USA). The gradient system was set up according to a previous report [32], with a slight modification to methanol concentration gradient. The pigment was monitored at 600 nm with a running time of 20 min and then compared with the standard.

### Determination of total phenolic content (TPC) and antioxidant activity

TPC was determined calorimetrically using a modified Folin-Ciocalteau (FC) method [39]. The cell-free broth (0.5 mL) was added to 0.2 mL of FC reagent, and the sample was allowed to stand at room temperature for 10 min. Thereafter, 0.6 mL of 20% sodium carbonate was added to the mixture, followed by incubation at 40°C for 30 min. The absorbance measurement was performed at 765 nm. Deionized water was used as the blank, and FC reagent was used as the control. Gallic acid was used as a standard to generate a calibration curve, and the TPC was expressed as grams of gallic acid equivalents per liter.

The antioxidant activity was evaluated by using a DPPH free-radical-scavenging assay according to a previous report [40]. The cell-free broth (0.5 mL) was mixed with 1.0 mL of 0.1 mM DPPH. The decolorization of DPPH was measured as the decrease in absorbance at 517 nm. Trolox (6-hydroxy- 2,5,7,8-tetramethylchroman-2-carboxylic acid) was used as a standard. The DPPH free-radical-scavenging activity was calculated as follows:

$$Scavenging\;activity\;\left(\%\right)=\left[1-\left(\frac{A1-A2}{A0}\right)\right]100$$

where A0 is the light absorbance of the DPPH control sample, A1 is the light absorbance of the reaction mixture sample, and A2 is the light absorbance of the sample with deionized water (no DPPH). The half- maximal inhibitory concentration (IC_50_, μg/mL) was determined from the plot of DPPH-scavenging activity against InK concentrations.

### Image analysis of fungal morphology

Using a bright-field microscope, the mycelial morphology and intracellular pigment distribution of the AoInK culture treated with Tween 80 were compared with those of the untreated culture. Fungal mycelia that were grown for 1, 3, and 5 d were image analyzed using an Olympus BX53/DP73 microscope coupled with licensed CellSens Standard software. A mycelial sample from the recipient (non-pigmented strain) was used as a control.

### Statistical analysis

Statistical analysis was performed using SPSS version 11.5 software for Windows (SPSS Inc., USA). Each experiment was performed in triplicate. The results are presented as mean values with standard deviation (mean ± SD). The data were subjected to one-way analysis of variance (ANOVA), and compared using Duncan’s multiple range test (DMRT) with statistically significant difference (*p-*value *<* 0.05).

## Results

### Overexpression of the indigoidine synthetase gene in *A*. *oryzae*

**Fig 1:**
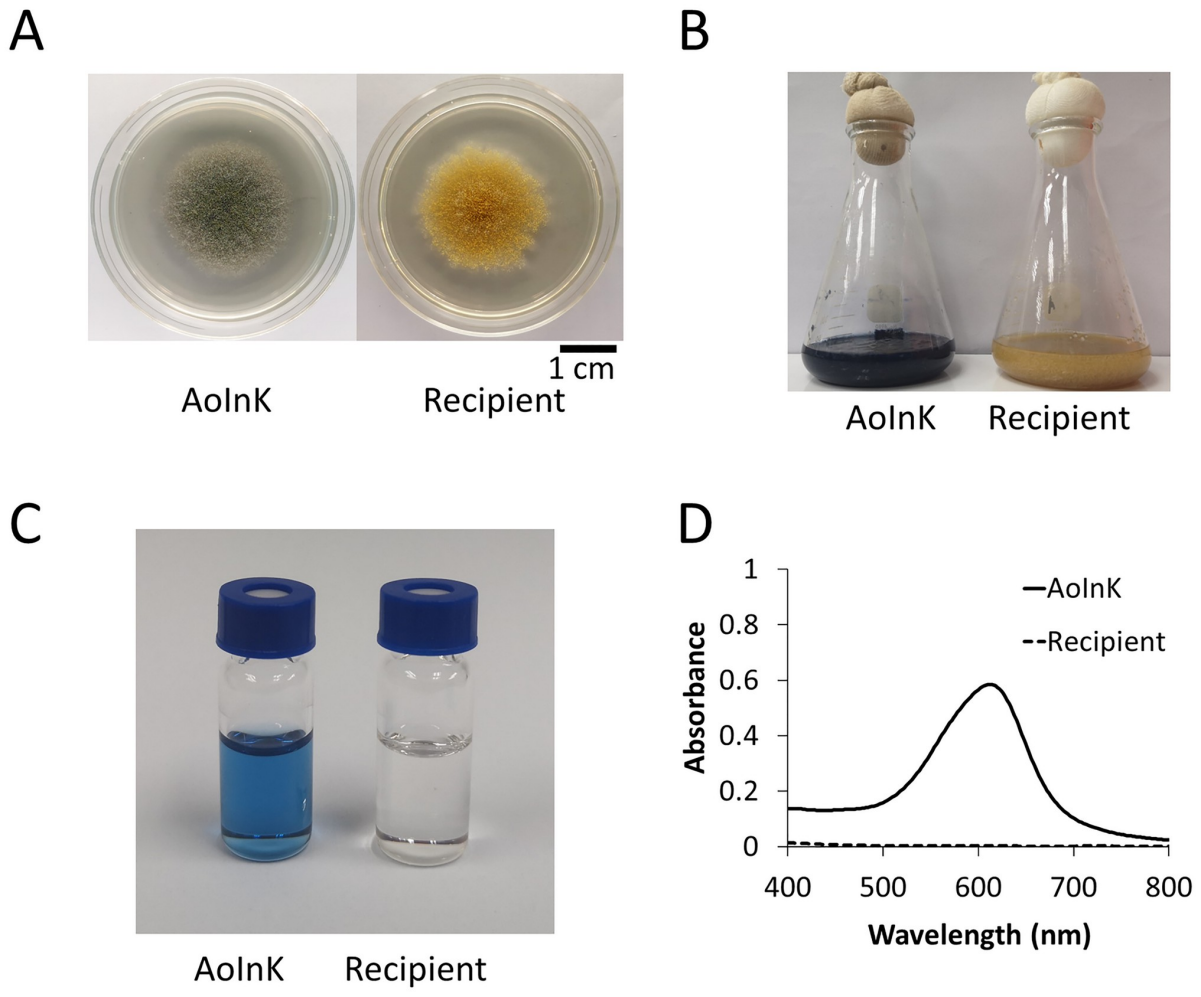
Phenotypic characteristics of the engineered AoInK strain and InK production. Colony growth of the engineered strain (left) and recipient (right) on Czapek Dox agar for 5 d (A). Submerged cultures of the engineered strain with dark blue color (left panel) and the recipient as a control (right panel) (B). The color of DMSO extract of AoInK mycelia (left panel) in comparison with that of the control (right panel) (C). Ultraviolet-visible spectrum of the indigoidine extract of the AoInK strain (D).

The 4134-bp codon-optimized sequence (*AoinK*) designed from the *ScindC* was functionally overexpressed in *A*. *oryzae* under the constitutive control of *AnPgpdA* promoter, generating the AoInK strain. The functional activity of the InK multi-functional synthetase was clearly observed as a dark blue colony of the engineered strain grown on solid agar, as shown in Fig 1A (left panel). After submerged fermentation, the fungal culture appeared dark blue, as shown in Fig 1B (left panel), in contrast to the control (recipient strain). A clear brilliant blue solution of DMSO extract of mycelial cells of the AoInK strain was also observed, as shown in Fig 1C, exhibiting a spectral peak with visible light absorption at 612 nm (Fig 1D. RP-HPLC analysis indicated that the indigoidine pigment was detected only in the AoInK culture, as shown in S2 Fig. Pigment production during growth of the engineered strain in the basal SM medium showed that the total InK titer increased with the increase in biomass titer. The maximal titer (287 mg/L) was obtained in the 5-d culture, in which glucose was exhausted (S3 Fig).

### Indigoidine production from the AoInK strain at different cultivation conditions

**Fig 2:**
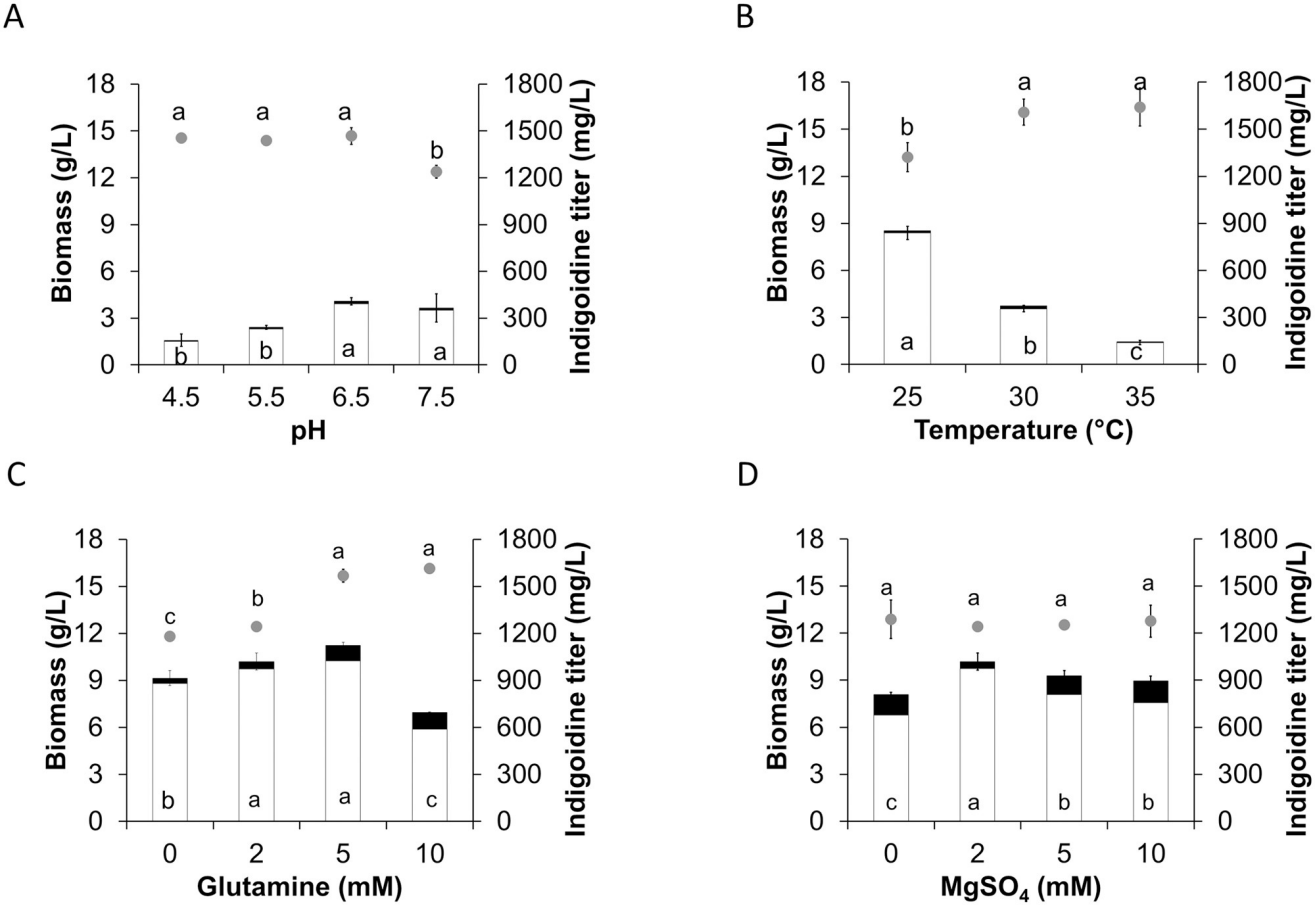
Effect of cultivation conditions on cell growth and InK production of the AoInK strain. All fungal cultures were grown in selective medium (SM) with agitation at 200 rpm for 5 d. Cultures grown with varying pH values at 30°C (A). Fungal cultures grown at initial pH 6.5 at varying temperatures (B). Fungal cultures grown at initial pH 6.5 and then supplemented with varying concentrations of glutamine and incubated at 25°C (C). Fungal cultures supplemented with 2 mM glutamine, grown at initial pH 6.5, and supplemented with varying MgSO_4_ concentrations (D). Dry biomass (gray circle) and intracellular and extracellular InK titers (white and black bars, respectively) are shown. Letters above the symbol and inside the bars indicate the significant difference in dry biomass and total InK titers (*p* < 0.05), respectively.

At different initial pH conditions for fungal cultivation, a dark blue pigment was observed in all cultures of the engineered strain of *A*. *oryzae*. The optimal condition for total InK production by the AoInK strain was pH 6.5–7.5, yielding an indigoidine titer of 365–408 mg/L, which is approximately 34–41% higher than the titer of the culture with an optimal pH of 5.5 for biomass production (241 mg/L indigoidine) as shown in Fig 2A. At initial pH 6.5, the InK titer of the AoInK culture was significantly improved when grown at 25°C, showing a two-fold increase (853 mg/L) compared to the pigment production at 30°C, as shown in Fig 2B. However, the InK titer decreased markedly when grown at 35°C.

Glutamine is a direct precursor for the biosynthesis of InK. The effect of glutamine supplementation on pigment production in the engineered strain was investigated by growing the fungus in a basal medium (pH 6.5) at 25°C. Although InK was generated in the culture without glutamine supplementation, pigment production was significantly enhanced by the addition of optimal concentrations of glutamine (2 and 5 mM), as shown in Fig 2C. Although biomass production was enhanced with increase in glutamine concentrations, an overly high glutamine concentration (10 mM) negatively contributed to the InK titer.

Magnesium (Mg^2+^) is a co-factor involved in the conversion of INDS to its active form. The concentration of this divalent cation may affect pigment production by the AoInK strain. The fungal cultures were supplemented with various MgSO_4_ concentrations. The results of experiments with varying MgSO_4_ concentrations showed that there was not much difference in biomass titers among the cultures with different MgSO_4_ concentrations. However, the addition of 2 mM MgSO_4_ was sufficient for pigment production, as clearly illustrated by the intracellular indigoidine titer (Fig 2D). Notably, a substantial amount of indigoidine accumulated in the fungal cells, with the ratio of intracellular and extracellular indigoidine concentrations varying from 5:1 to 24:1, depending on the cultivation conditions (Fig 2).

### Effect of nonionic surfactants on extracellular production of indigoidine

**Fig 3:**
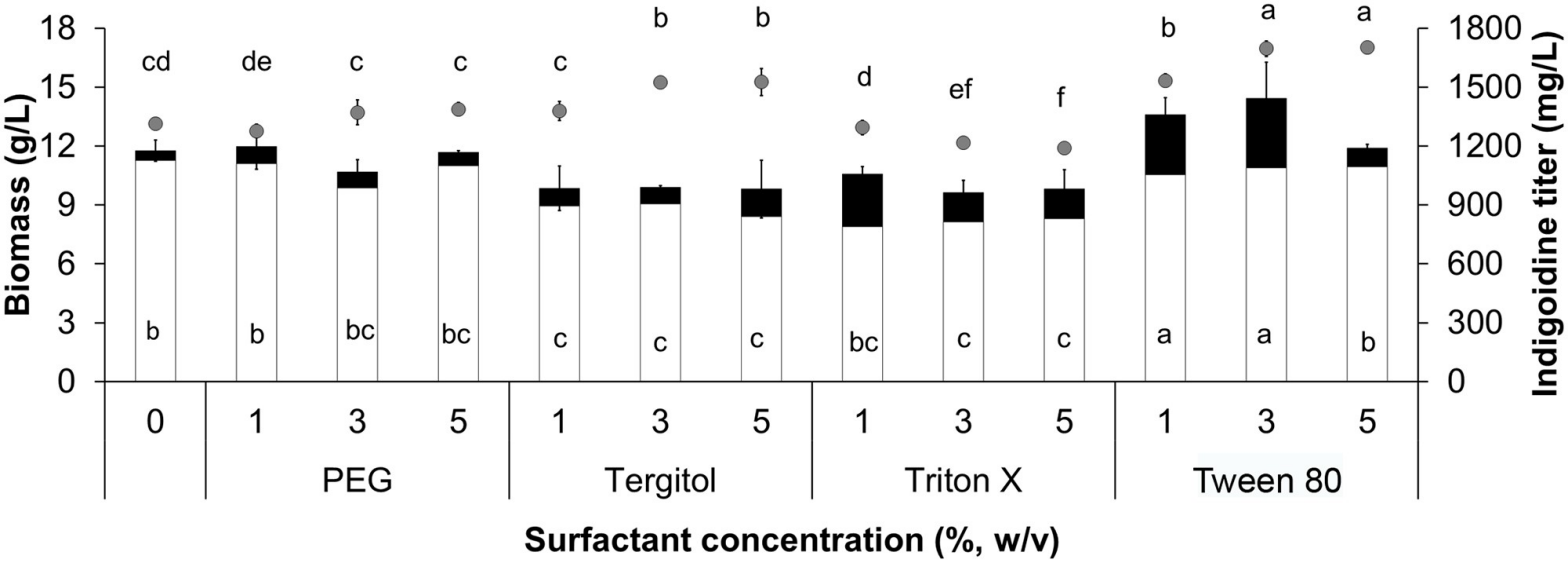
Cellular growth and extracellular InK production of AoInK cultures treated with nonionic surfactants. Dry biomass (gray circle) and intracellular and extracellular InK titers (white and black bars, respectively) of the AoInK cultures treated with different nonionic surfactants. Letters above the symbol and inside the bars indicate significant difference in dry biomass and the total InK titers (*p* < 0.05), respectively.

The effect of nonionic surfactants on InK pigment secretion by the AoInK cells was studied. As shown in Fig 3, the extracellular InK titers of the AoInK cultures with 2 mM glutamine were enhanced by all the nonionic surfactants that were evaluated. Triton X-100 and Tween 80 solutions could promote extracellular InK titers compared to other surfactants. The amount of secreted InK was dependent on the concentration of nonionic surfactants. High concentration of Triton X-100 (3% and 5%) negatively affected total pigment (intracellular and extracellular indigoidine) titers and biomass, whereas the maximal biomass and total InK titers were detected in the fungal culture treated with 3% Tween 80 (Fig 3). Consequently, a 5–6-fold increase in extracellular pigment was achieved by the addition of 1% and 3% Tween 80 solution as compared with the untreated culture. Supplementation with Triton X-100 at a low concentration (1%) also induced extracellular pigment secretion from mycelial cells of the AoInK strain.

**Fig 4:**
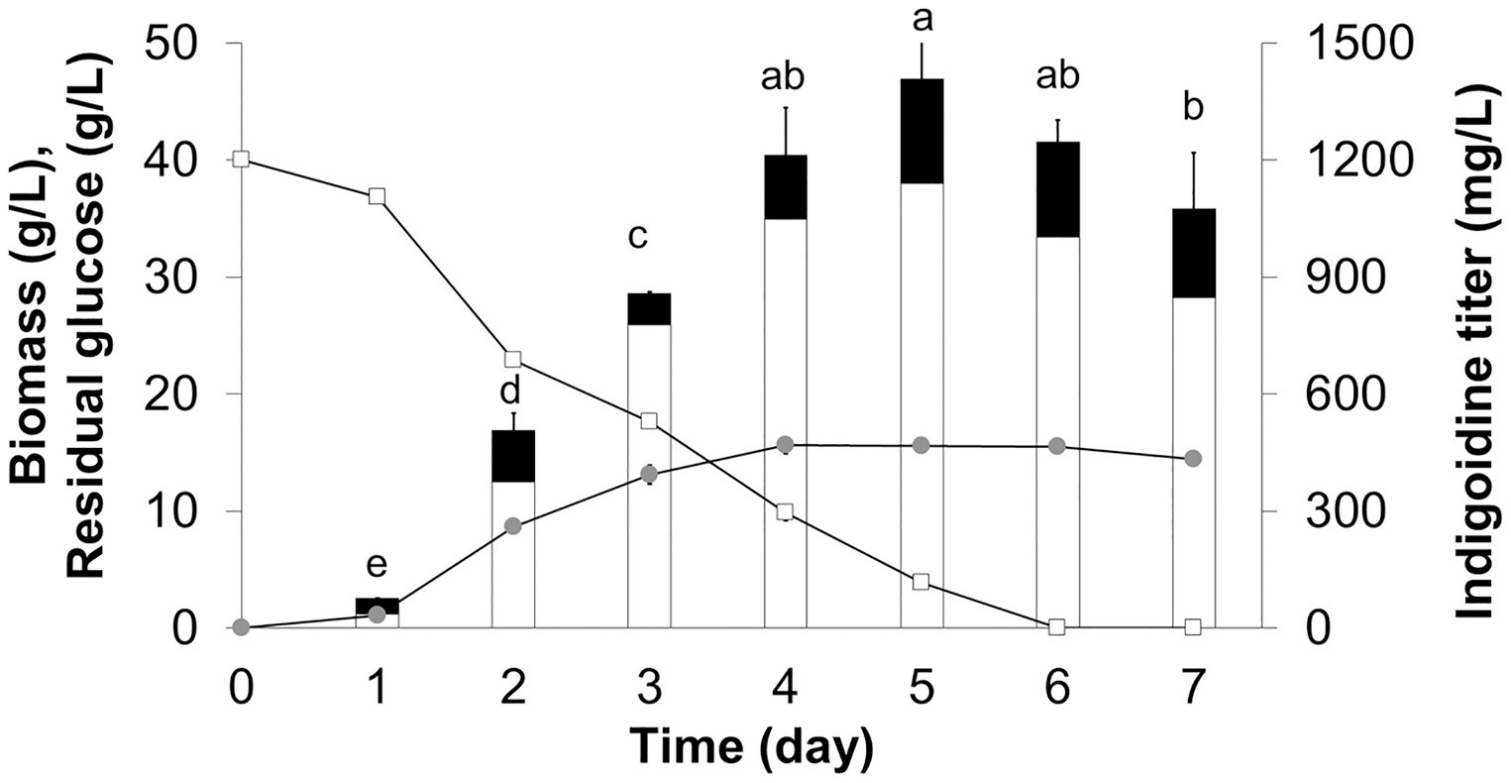
Cellular growth and extracellular InK production profiles of AoInK cultures treated with Tween 80. Fungal cultures were grown in SM medium supplemented with 2 mM glutamine and 1% Tween 80 at 25°C, 200 rpm. The samples were taken at different cultivation times for analyses. Dry biomass (gray circle), residual glucose concentration (white square), and intracellular and extracellular InK titers (white and black bars, respectively) are shown. Letters above the bars indicate significant differences in total InK titer (*p* < 0.05).

The cell growth and pigment production profiles of the AoInK strain were analyzed. When cultures were supplemented with 2 mM glutamine and 1% Tween 80, the total indigoidine titer increased with the increase in cultivation time. The maximal level of total indigoidine (1409.22 ± 95.33 mg/L) was found at the end of the growth phase (5-d cultivation, Fig 4), indicating that this metabolite was growth associated. The biomass titer of the engineered strain was lower than that of the recipient strain. However, the growth profiles of both cultures were similar (S4 Fig). The pigment production yields of the AoInK culture supplemented with 2 mM glutamine and 1% Tween 80 were 37.66 ± 1.23 mg/g glucose and 90.29 ± 5.63 mg/g dry biomass which were 4–5-fold higher than those of the basal SM culture (7.17±1.20 mg/g glucose and 20.36±1.99 mg/g dry biomass, respectively). Moreover, the InK productivity of the culture grown under the optimized condition was 265.09 ± 14.74 mg/L·d which was also an approximate 4–5-fold increase when compared to culture grown in basal SM medium (57.37 ± 9.58 mg/L·d).

### TPC and antioxidant activity of InK from the AoInK strain

**Fig 5:**
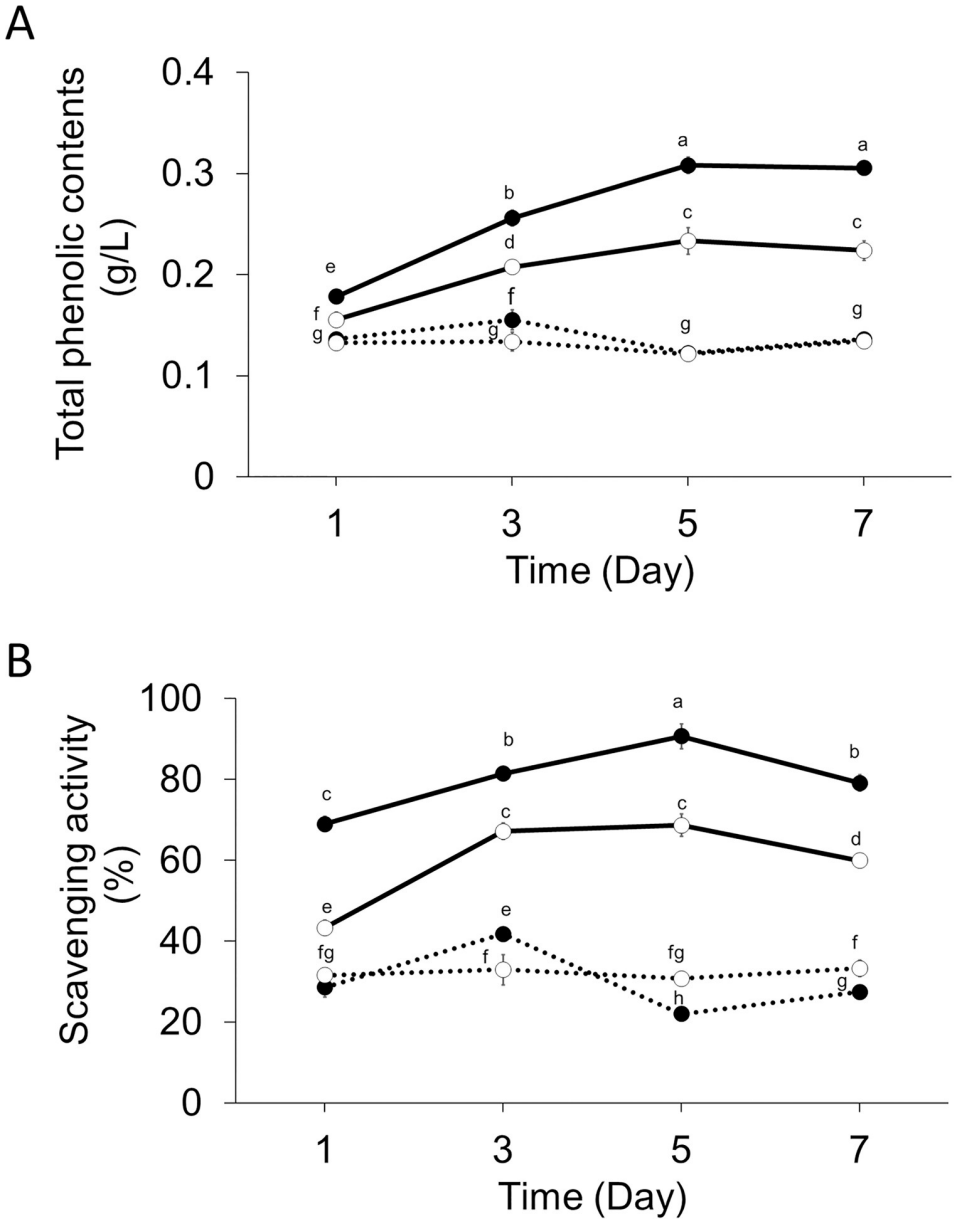
Total phenolic content and antioxidant activity of InK derived from the engineered strain. Total phenolic content of InK in cell-free broth of AoInK cultures grown at different cultivation times (A). Scavenging activity of InK (B) in cell-free broth of AoInK cultures grown at different cultivation times. Smooth and dotted lines indicate the cultures of AoInK and recipient strains, respectively. Black circles represent 1% Tween-treated cultures; white circles represent untreated cultures. Letters above the circles indicate the significant differences in measured values (*p* < 0.05).

With regard to the proposed antiradical and antioxidant properties of InK, as described above, the TPC and antioxidant activity of the 1% Tween 80-treated and untreated cultures of the AoInK strain were investigated. Results of the investigation into the antiradical and antioxidant properties based on the structural features of InK showed that the TPC of both cultures increased with the cultivation time (Fig 5A). The TPC of the Tween 80-treated culture was higher than that of the culture without the nonionic treatment, which corresponded to the pigment titers. The highest TPC (0.31 ± 0.01 g/L) was obtained in the Tween 80-treated culture grown for 5 d. In contrast, an extremely low TPC was measured for the recipient strain, which was unable to produce InK. The pigment that was extracted from the 5-d culture with Tween 80 treatment showed the highest scavenging rate (90.68 ± 3.07%, Fig 5B). The IC_50_ value representing the concentration of InK that caused 50% neutralization of DPPH radicals was 38.89 ± 1.56 μg/mL (S5A Fig); this value was closely related to the value of the indigoidine standard (39.20 ± 0.06 μg/mL, S5B Fig). These results suggest that the recombinant InK produced by the AoInK strain showed antioxidant activity and that the inhibitory scavenging efficiency of this pigment was reliable for the commercial InK product.

### Image analysis of the indigoidine-producing strain of *A*. *oryzae*

**Fig 6:**
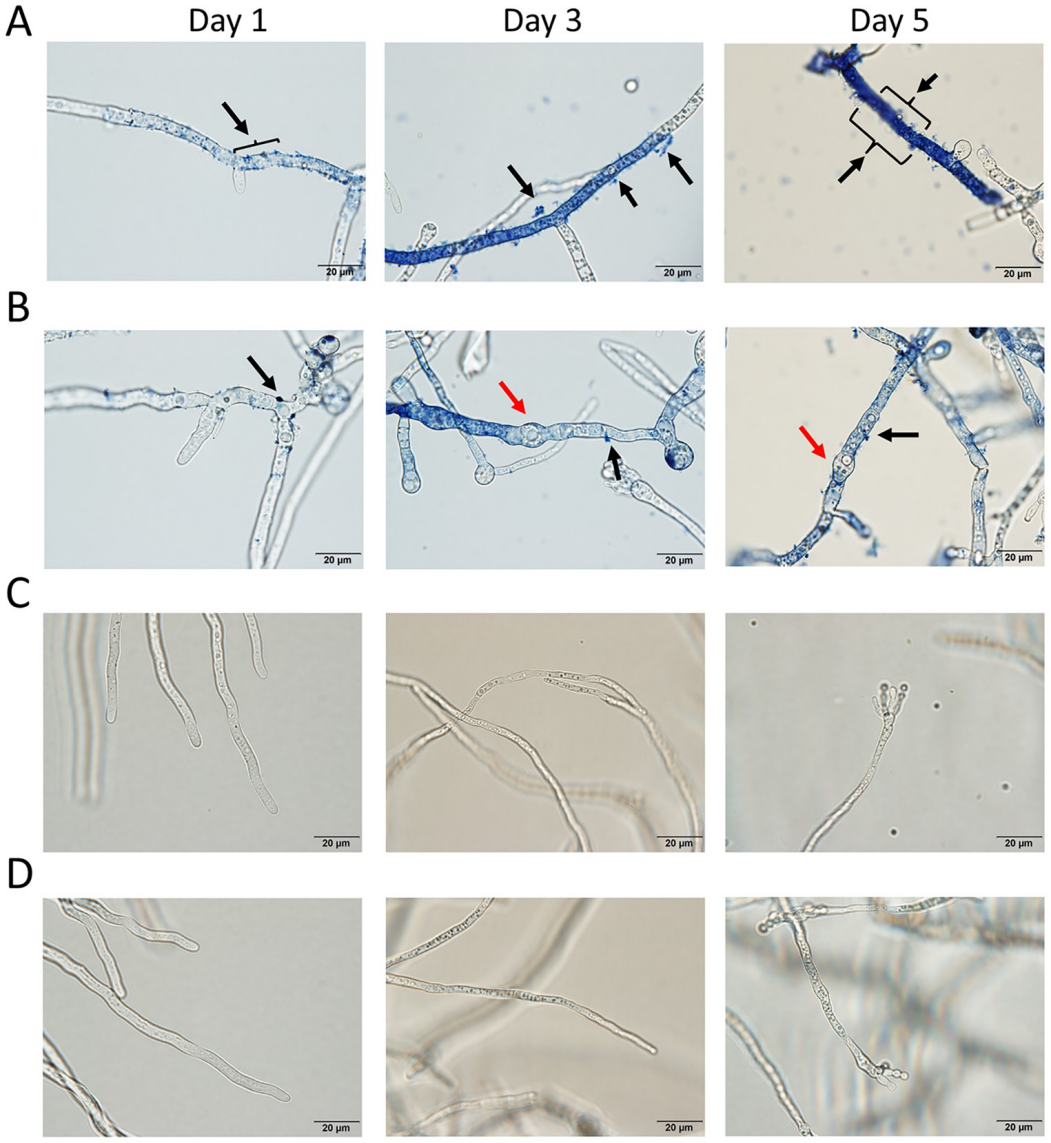
Image analysis of the mycelia of AoInK cultures treated with 1% Tween 80. Mycelial cultures of AoInk and recipient strains were grown at 25°C, 200 rpm, and the samples were taken at different cultivation times for image analysis using the Olympus BX53/DP73 bright field microscope (100× magnification). Tween 80-treated mycelia of the AoInK strain (A). Untreated mycelia of the AoInK strain (B). Black arrows indicate blue pigment granules attached to cell surface, and red arrows indicate swollen hyphae containing vacuoles. Tween 80-treated mycelial cells of the recipient culture (C). Untreated mycelial cells of the recipient culture (D). Data represent three independent experiments.

Through image analysis under a bright field microscope, blue pigment granules were observed in the mycelial cells of AoInK strain in both Tween 80-treated and untreated cultures (Fig 6). High pigment accumulation, as illustrated by color intensity, was clearly detected in the 1% Tween 80-treated culture grown for 5 d (Fig 6, panel A), when compared with the untreated culture (Fig 6, panel B). The hyphal surface was attached by dark blue granules (black arrows), particularly in the AoInK culture with 1% Tween 80 treatment. This result indicated that pigment secretion occurred from the mycelial cells along the growth stages, which coincided with the InK titers, as shown in Fig 4. It seems that secretion might occur from the sub-apical hyphae (Fig 6A and 6B, day 1). Swollen hyphae with vacuoles inside (red arrows) were observed only in the pigment-producing strain without Tween 80 treatment (Fig 6B, days 3 and 5), but were not detected in the Tween 80 treatment throughout the cultivation period (Fig 6A).

## Discussion

The NRPS system has been discovered in diverse microbial species through the genetic evolution of bacteria to higher eukaryotes via horizontal gene transfer [41–43] and is involved in cell growth and adaptation [44]. Recently, the development of oligopeptide production in *A*. *oryzae* using the NRPS system has been reported, exploring the potential of this complex system for gene editing in filamentous fungi [32]. In this study, the *nrps* gene (*ScindC*) encoding the mega-synthetase for indigoidine biosynthesis was chosen as the target for indigoidine overexpression in *A*. *oryzae*. The engineered strain was able to synthesize the dipeptide pigment during cultivation, verifying the functional NRPS machinery of this fungus to support foreign dipeptide pigment production. However, the concomitant increase in InK and biomass titers during cell growth (Fig 4) indicated that this dipeptide pigment was a growth-related metabolite in the AoInK strain. This was a result of constitutive gene expression under the control of *AnPgpdA* promoter. The intracellular pigment product might slightly affect cell growth, as indicated by the low biomass titer compared with the recipient (S4 Fig). The dipeptide is small and does not form misfolded structures or toxic aggregates when compared to high-molecular weight proteins [45]. This suggests that the dipeptide pigment metabolite does not have a direct negative impact on fungal growth. Furthermore, the chemical structure of the pigment indicates its involvement in free radical scavenging. The presence of hydroxyl and/or amino group(s) in phenolic, enolic, or anilinic compounds (as presented in the InK structure) has been proven for hydroxyl-radical-scavenging activity [12]. Our results verified the biological function of the recombinant pigment produced by the engineered strain of *A*. *oryzae* and showed that the antioxidant activity was retained.

The production of dipeptide pigment in *A*. *oryzae* can be improved through physiological manipulation. In fact, the optimal cultivation conditions for heterologous production of targeted metabolites are species dependent, underlying the metabolic regulation in host cells. Usually, acidic conditions are preferred for *A*. *oryzae* growth [46] but not for indigoidine production by the AoInK strain, in which the ionic balance might contribute to the activities of enzymes involved in pigment biosynthesis. Comparable result has been previously reported for *Penicillium purpurogenum* [47]. The significant effect of low temperature on the InK titer of the engineered strain may be explained by the fact that regulation of fungal secondary metabolism is reliable for low-temperature-responsive transcriptional factors and biosynthetic enzyme stability [48]. The supplementation of high glutamine concentrations in the culture might shift the metabolic flux toward biomass production rather than pigment biosynthesis. We suggest that glutamine should be allocated at an optimal level to maximize the InK production yield. The role of a divalent cation agent (Mg^2+^) in pigment production by the AoInK strain was also explored. We found that Mg^2+^ is involved in the regulation of secondary metabolism, consistent with a previous report [49]. Mg^2+^ is required for 4ʹ-PPTase activity to convert inactive INDS to the active form for metabolite biosynthesis. We postulate that magnesium is a co-factor ion for the activation of INDS, based on our finding that the optimal concentration of magnesium (2 mM) was required to enhance pigment production by the engineered strain. A magnesium concentration lower than the optimal level might be insufficient for 4ʹ-PPTase activity, and reduced production may result from the feedback inhibition of phosphopantetheinylation, when the magnesium concentration is excessive.

Although the blue pigment mostly accumulated in the cells, its secretion corresponding to the increase in total InK content might be attributed to overproduction, particularly in the 5-d culture. In filamentous fungi, the mechanism controlling metabolite secretion, particularly that of secondary metabolites, has not been elucidated. A scheme for secondary metabolite production and secretion by filamentous fungi has been previously reported [50]. Studies have proven that the secondary metabolite biosynthesis particularly in filamentous fungi is generated in endosomes or peroxisomes, wherein synthetases and other tailor-made enzymes exist. Apart from the transport system via plasma membrane transporters, the secretion of certain metabolites can be coupled with the classical secretory route through exocytosis, as reported for yeasts and filamentous fungi [25, 51, 52]. It is likely that the InK secretion found in the engineered strain of *A*. *oryzae* might be involved in exocytosis, as observed when the pigment was overproduced (Fig 6). The dark blue protrusions observed in the engineered strain of *A*. *oryzae* might be a result of the attachment of InK to the cell surface, similar to a previous report describing the secretion mechanism of aflatoxin and pathogenicity-related effectors via exocytosis [52–54].

Modification of the cell membrane structure is an alternative strategy to enhance production by releasing the generated protein/enzymes across the membrane. Nonionic surfactants play a role in cell membrane integrity and permeability [55, 56]. Our study demonstrated that Tween 80 enhanced total pigment production (Fig 3), which might be explained by the dual action of the nonionic surfactant. Oleic acid (C18:1 *n*-9), which is a constituent of Tween 80, is an alternative carbon source to support growth of the AoInK strain, thus leading to pigment overproduction [57, 58]. The enhanced pigment secretion observed was also a result of the amphipathic characteristics of Tween 80, which was attributed to the permeability of the cytoplasmic membrane, as previously described [27, 59]. The structural features of InK do not appear to be toxic to fungal cells. However, the low biomass of the AoInK strain compared to that of the recipient might have resulted from the leverage of metabolic energy for cell growth and production of the colored dipeptide. Pigment secretion is also a cellular mechanism that sustains fungal growth and reduces stress, as shown by the morphological alteration of hyphae (Fig 6).

The results of this study present a potent approach to employing *A*. *oryzae* as a platform for producing indigoidine through synthetic biology. In contrast to some bacterial and yeast cell factories [16, 22], this fungal chassis cell has a complete NRPS system that permits a feasible option for efficient synthesis of the dipeptide pigment indigoidine. Biosynthesis can be achieved with minimal manipulation by the constitutive overexpression of the single *AoinK* gene, as proven in this study. Furthermore, the AoInK strain was cultivated in various conditions (varying pH, temperature, nutrient concentration, and nonionic surfactant treatment); thus, our findings reveal the robust features of the AoInK strain, facilitating bioprocess development with no behavioral trade-offs affecting the InK yield. Secretion system existing in this fungus is a promising machinery for the extracellular production of foreign pigments. We suggest that this fungal system is a promising platform for manufacturing bioactive pigments as well as other extracellular secondary metabolites, including NRP compounds, demonstrating a simple harvesting process and feasibility for large-scale industrial production.

## Supporting information

S1 Fig Plasmid map of pAoInK-pyrG containing the *AoinK* expression cassette and *pyrG* selectable marker (A), and enzyme restriction analysis of the recombinant plasmid (B). Lane 1 indicates the 2.3, 7.2 and 7.6-kb DNA fragments digested by *Eco*RI and *Not*I. Lane 2 indicates 0.9-, 6.7- and 9.4-kb DNA fragments digested by *Not*I and *Sgs*I. Lane M is Thermo Scientific GeneRuler 1 kb DNA Ladder.
(DOCX)

S2 Fig Analysis of indigoidine in the AoInK strain. Reversed-phase high performance liquid chromatography (RP-HPLC) analysis of indigoidine derived from the AoInK and recipient strains compared with the indigoidine (InK) standard. Arrows indicate chromatographic peaks of indigoidine with retention times (Peaks A and B).
(DOCX)

S3 Fig Profiling of cell growth and InK production of the AoInK culture grown in the basal SM medium. Fungal cultures were grown at 30°C with shaking at 200 rpm. Dry biomass (grey circle), residual glucose (white circle), intracellular and extracellular InK titers (white and black bars, respectively) are shown. Letters above the bars indicate significant difference in the total InK titers (*p* < 0.05).
(DOCX)

S4 Fig Profiling of cell growth of the AoInK and recipient strains. Fungal cultures were grown in the SM medium supplemented with 2 mM glutamine and 1% Tween 80 at 25°C, 200 rpm. The samples were taken at different cultivation times for analyses. Dry biomass titers of the AoInK (grey circle) and recipient (white circle) strains are shown. Residual glucose concentrations of the AoInK and recipient strains are represented by grey and white squares, respectively. Letters above the circles indicate significant difference in cell biomass of both strains (*p* < 0.05). The experiments were carried out in triplicates.
(DOCX)

S5 Fig Determination of IC_50_ values of InK derived from the AoInK strain (A) and the InK standard (B). The IC_50_ value was determined from the plot of DPPH scavenging activity (%) against InK concentrations.
(DOCX)

S1 Table The codon-optimized sequence of *AoinK* gene. (DOCX)

S2 Table Overlapping primer sets used for amplifying specific DNA fragments by PCR. (DOCX)
