# Supplementary material for: Potential of Aspergillus oryzae as a biosynthetic platform for indigoidine, a non-ribosomal peptide pigment with antioxidant activity
Source: PLoS One. 2022 Jun 23;17(6):e0270359. doi: 10.1371/journal.pone.0270359 (PMC9223385; doi:10.1371/journal.pone.0270359)
Supplement: S1 Fig — Lane 1 indicates the 2.3, 7.2 and 7.6-kb DNA fragments digested by EcoRI and NotI. Lane 2 indicates 0.9-, 6.7- and 9.4-kb DNA fragments digested by NotI and SgsI. Lane M is Thermo Scientific GeneRuler 1 kb DNA Ladder. (DOCX) [file pone.0270359.s001.docx]

| A  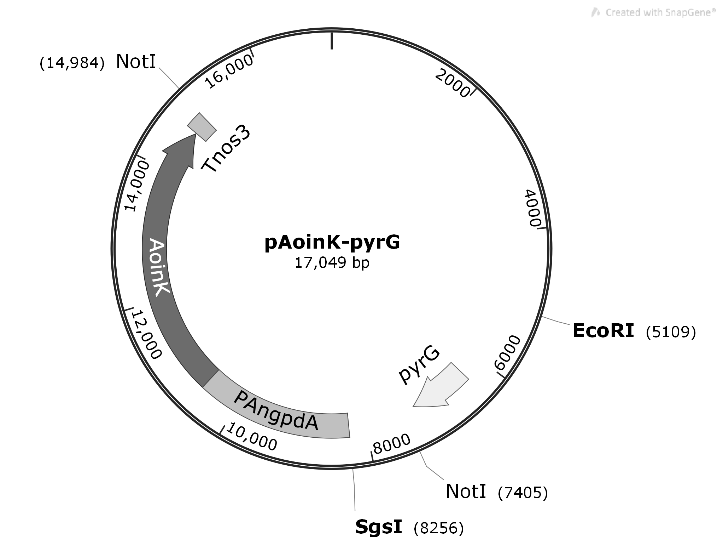 | B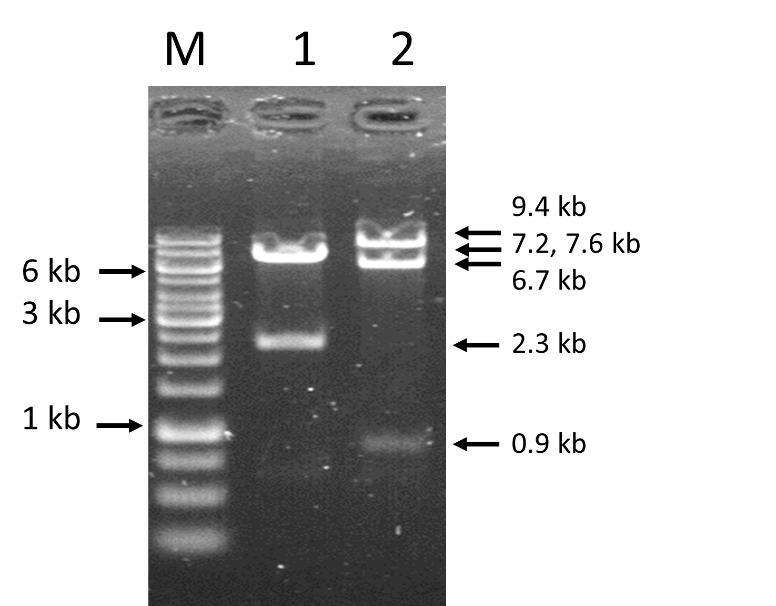 |
| --- | --- |
| S1 Fig. Plasmid map of pAoInK-pyrG containing the *AoinK* expression cassette and *pyrG* selectable marker (A), and enzyme restriction analysis of the recombinant plasmid (B).  Lane 1 indicates the 2.3, 7.2 and 7.6-kb DNA fragments digested by *Eco*RI and *Not*I*.* Lane 2 indicates 0.9-, 6.7- and 9.4-kb DNA fragments digested by *Not*I and *Sgs*I*.* Lane M is Thermo Scientific GeneRuler 1 kb DNA Ladder. | |
