# Supplementary material for: Potential of Aspergillus oryzae as a biosynthetic platform for indigoidine, a non-ribosomal peptide pigment with antioxidant activity
Source: PLoS One. 2022 Jun 23;17(6):e0270359. doi: 10.1371/journal.pone.0270359 (PMC9223385; doi:10.1371/journal.pone.0270359)
Supplement: S2 Fig — Reversed-phase high performance liquid chromatography (RP-HPLC) analysis of indigoidine derived from the AoInK and recipient strains compared with the indigoidine (InK) standard. Arrows indicate chromatographic peaks of indigoidine with retention times (Peaks A and B). (DOCX) [file pone.0270359.s002.docx]

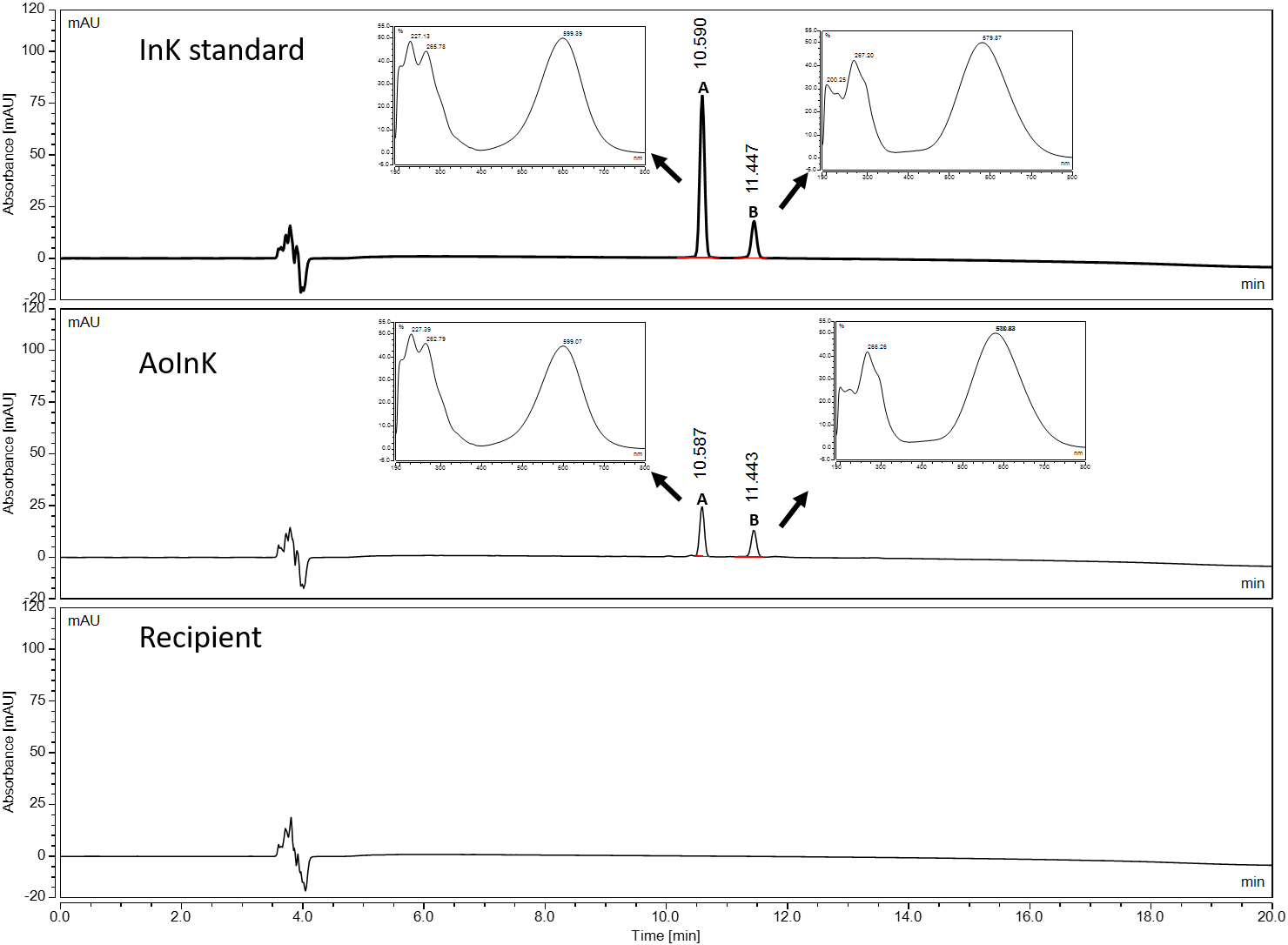


S2 Fig. Analysis of indigoidine in the AoInK strain.

Reversed-phase high performance liquid chromatography (RP-HPLC) analysis of indigoidine derived from the AoInK and recipient strains compared with the indigoidine (InK) standard. Arrows indicate chromatographic peaks of indigoidine with retention times (Peaks A and B)**.**
