# Supplementary material for: Potential of Aspergillus oryzae as a biosynthetic platform for indigoidine, a non-ribosomal peptide pigment with antioxidant activity
Source: PLoS One. 2022 Jun 23;17(6):e0270359. doi: 10.1371/journal.pone.0270359 (PMC9223385; doi:10.1371/journal.pone.0270359)
Supplement: S3 Fig — Fungal cultures were grown at 30°C with shaking at 200 rpm. Dry biomass (grey circle), residual glucose (white circle), intracellular and extracellular InK titers (white and black bars, respectively) are shown. Letters above the bars indicate significant difference in the total InK titers (p < 0.05). (DOCX) [file pone.0270359.s003.docx]

| 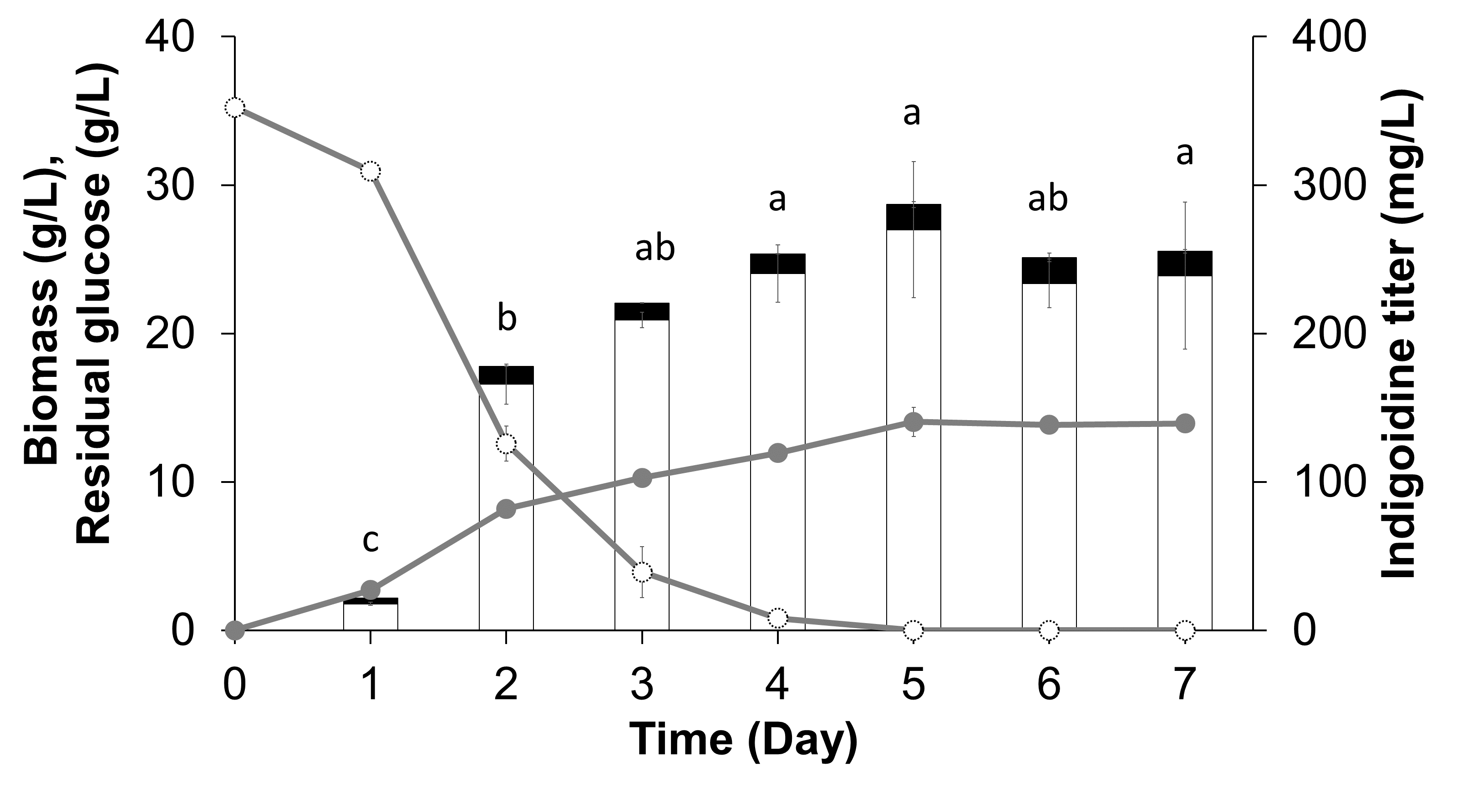 |
| --- |
| S3 Fig. Profiling of cell growth and InK production of the AoInK culture grown in the basal SM medium.  Fungal cultures were grown at 30°C with shaking at 200 rpm. Dry biomass (grey circle), residual glucose (white circle), intracellular and extracellular InK titers (white and black bars, respectively) are shown. Letters above the bars indicate significant difference in the total InK titers (*p* < 0.05). |
