# Supplementary material for: Potential of Aspergillus oryzae as a biosynthetic platform for indigoidine, a non-ribosomal peptide pigment with antioxidant activity
Source: PLoS One. 2022 Jun 23;17(6):e0270359. doi: 10.1371/journal.pone.0270359 (PMC9223385; doi:10.1371/journal.pone.0270359)
Supplement: S4 Fig — Fungal cultures were grown in the SM medium supplemented with 2 mM glutamine and 1% Tween 80 at 25°C, 200 rpm. The samples were taken at different cultivation times for analyses. Dry biomass titers of the AoInK (grey circle) and recipient (white circle) strains are shown. Residual glucose concentrations of the AoInK and recipient strains are represented by grey and white squares, respectively. Letters above the circles indicate significant difference in cell biomass of both strains (p < 0.05). The experiments were carried out in triplicates. (DOCX) [file pone.0270359.s004.docx]

| 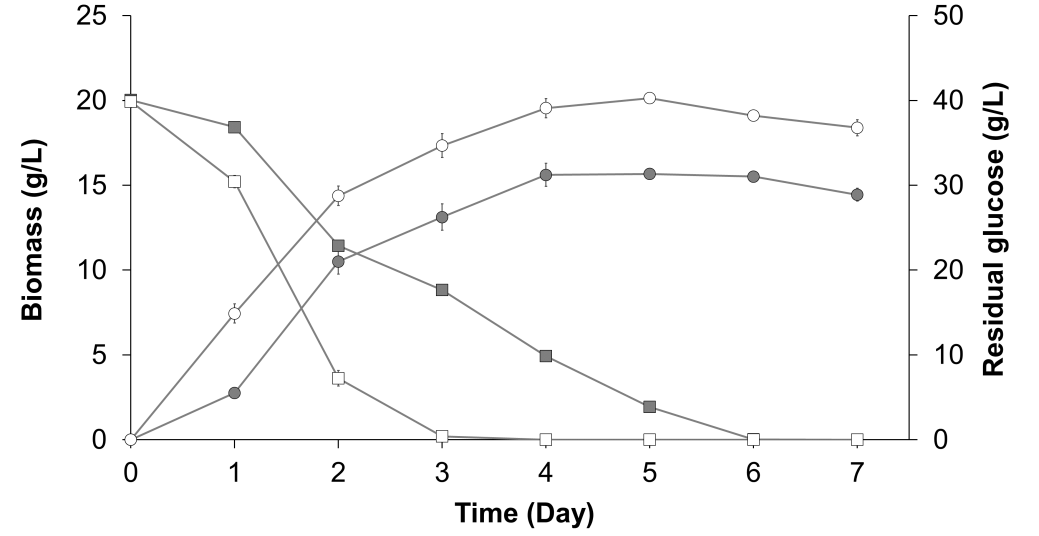  c  a  a  ab  ab  f  g  e  d  cd  cd  c  c  b |
| --- |
| S4 Fig. Profiling of cell growth of the AoInK and recipient strains.  Fungal cultures were grown in the SM medium supplemented with 2 mM glutamine and 1% Tween 80 at 25°C, 200 rpm. The samples were taken at different cultivation times for analyses. Dry biomass titers of the AoInK (grey circle) and recipient (white circle) strains are shown. Residual glucose concentrations of the AoInK and recipient strains are represented by grey and white squares, respectively**.** Letters above the circles indicate significant difference in cell biomass of both strains (*p*<0.05). The experiments were carried out in triplicates. |
