# Supplementary material for: Potential of Aspergillus oryzae as a biosynthetic platform for indigoidine, a non-ribosomal peptide pigment with antioxidant activity
Source: PLoS One. 2022 Jun 23;17(6):e0270359. doi: 10.1371/journal.pone.0270359 (PMC9223385; doi:10.1371/journal.pone.0270359)
Supplement: S5 Fig — The IC50 value was determined from the plot of DPPH scavenging activity (%) against InK concentrations. (DOCX) [file pone.0270359.s005.docx]

| \| **A)** \| **B)** \| \| --- \| --- \| \|  \|  \| \| S5 Fig. Determination of IC_50_ values of InK derived from the AoInK strain (A) and the InK standard (B).  The IC_50_ value was determined from the plot of DPPH scavenging activity (%) against InK concentrations. \| \| |
| --- | --- | --- | --- | --- | --- | --- |
