# Supplementary material for: Potential of Aspergillus oryzae as a biosynthetic platform for indigoidine, a non-ribosomal peptide pigment with antioxidant activity
Source: PLoS One. 2022 Jun 23;17(6):e0270359. doi: 10.1371/journal.pone.0270359 (PMC9223385; doi:10.1371/journal.pone.0270359)
Supplement: S1 Table — (DOCX) [file pone.0270359.s006.docx]

S1 Table. The codon-optimized sequence of *AoinK* gene.

| **Gene** | **Sequence (5’🡪 3’)** |
| --- | --- |
| *AoinK* | ATGAGCGTTGAAACCATTCCTTGTAGCAGGCGTGCGGCTTTGGGACTTCCAGGGCTTTTGCGTGAGCGTGCCAGA  GCGACACCCGACCGCACCGCCGCCGTGCATGAACACCAGTCCCTCACTTTCGCCCAGCTGACTGAGGATTCGTCG CATGTTGGAGCCCTGCTCCGGCAGGCTGGTGTGGGACGGGACTCCCGTGTGGGTGTGTTCATGGAACCCTCCCTG GACCTGTTGACTGGTGTCTGGGGTATCCTGTGGGCCGGAGGTTGCTATGTGCCGCTGTCCCCCGAGTATCCCGAG GAACGGATCGCCTACATGCTGGCTGACGCCGGTGTGGACATCGTCCTCACTCAGGAGTTCTTGCGCAGCACCCTT CAGGAGTTGGCTCCAGCCGGAGTTGTGGTGCTGACCCTGGATGAGATGCTCCGTACTGCCGAACGTGACGGTTCG GCCTTCGGTCGGCCAGAACCCGAGGTGCGGCCCGATGACCTCGCTTACGTGATCTACACCTCTGGCTCCACCGGA AAGCCAAAGGGCGTGATGGTCGAGCACCGGTCGATCGTTTCCCAGATGCGGTGGCTCCACGATGAATGTGGTATC GATGAGAACGAGATCATCCTGCAGAAGACCCCAATGTCCTTCGATGCTGCCCAGTGGGAGCTCCTGGCCCTCGCC TGCGGTTCCACTGTCGTCATGGGATCGAGCGGTATCTATCGGGACCCGGAGGCTATCATCTCGACTGTGCAGCGG CACGGTGTCACCACTCTCCAGTGCGTCCCCACTCTCCTCCAGGCCCTGCTCGATACCGAAAAGTTCCCGGACTGT GGTACTCTGCGTCGGATCTTCTCGGGTGGAGAAGCCCTCTCCCGGTCGTTGGCCGCTCAGTGTTTGGATACCATG CCGGGCGCTCGGCTGGTTAACCTGTACGGACCTACTGAGTGCACTATCAACGCTTCCTCGTTCGTGGTCGATCGC GCTGCTCTCGAAGATGGACCACTCGTGATGCCCATCGGCACTCCAGTGCACGACACCTCGCTTCACGTGCTGCGT CCAGACGGCGCTCCCGTCAGCGCCGGTGAAATCGGCGAGCTGTATATCGGCGGTGTGCAGGTGGCTCGGGGCTAC CTCGGCCGTCCAGATCTCACTGGAGACCGGTTCATGGCCGACCCATTCAGCGATGCTCCAGGTTCGCGCCTCTAT CGGACTGGTGACCTCGCCCACGTCAACGCCGACGGAACCGTGCAGTTCGTTGGACGGGCCGATAACCAGGTGAAG TTGCGGGGTTACCGGGTGGAGCTTGATGAGATCCGGCAGACCGTGGAAACTCACGATTGGGTGCGCGCTGCCGCT GTCCTGCTGCGTGACGATGCTACCACTGGCTTCCAGAACCTGGTCGCTTTCGTTGAGCTCAATCCAAAGGAAGCT GCCCTTATGGATCAGGGTAACCACGGCTCTCACCACCAGTCCAAGGCTTCGCGGCTCCAGGTGCGCGCTCAGCTC GCTCATCCAGGTTGTCGGGACGATGCTGATCTGGCTGGACGTGCCGCTATCGACCTCCCAGGAGCTGAAGCCACT CCTGGTCAGCGGGCTCTTGCCTTCTCCCGGAAGACTTATCGGTTCTATGAAGGCTCGCCTGTCACCCGCGACGAC ATCCTGCACCTCCTTGGCCCCCGTCCACGGCCCCGTCCCTCGGCCCGTACCAGCGACATCGTGGGTCGGGATGAA CTGGGAACCATCCTGCGGAATTTCGGTCGGCACCTGTCGGACCAGCGTCTGCTCCCAAAGTATGCTTACGCCTCG CCAGGTTCGTTGTACGCCACCCAGCTCTATGTCGAGATCGGCGGTGGTCACGACGTTCCTGCTGGATTGTACTAT TATCATCCTCTTCACCACCGGTTGGTCCTCGTGGGACCAGCCTCTGAGACCGAGACTAGCCCGGTTCGCATCCAT TTCCTGGGTAAGCATGGAGCTATCGAACCAGTGTACCGGAACAATGTGCGGGAGGTGTTGGAAATCGAGGCCGGA CACATGGTGGGACTGTTCGAAGAGGTTCTCCCAGCTCATGGTCTTCGCATCGCCGCTGCCGCCTATCAGCCAGCT GTTCGTCATCGGCTCGATTGTGCTCCCGAAGATCATTACCTGGGTTCGTTCGATTTGCTGCCCCAGGCCCGTGGA GCTTCGGAGGATACTGATACTCTCGACATCTACGTGCAGGCTCACTCGACTCGGATCGAGGGTCTTCCACCCGGC CAGTACCGCTACACTGGTGCTGGCCTGGTGCGGATCGGAGACGATGTTATCCTCAAGAAGCATGTGATCGCTATC AATCAGCGGGTGTACGAACGCTCGGATTTCGGTATCTCCCTTGTCGCCACCGGTTCCGCCAGCTGGCGGCGTTAT CTGGACCTCGGACGCGGACTTCAGCGTCTGCAGATGAATGATCTGCATCTCGGTTTCATGTCCTCCGGTTACTCG TCTAAGTCGGGTAACGACCTGCCATCCGCCAAGCGTCTGGGACGTATCCTGGCCGACGGTGGTTTGCCCGCTGGT CCTTCGTATTTCTGCGTGGGTGGCCGGGTCTCTGATGCTCAGTGGCGGGGTGAGGACATGAAGGAAGACGTGGTC CACATGCAGGGTCCCGCTGAGCTCATCAAGGAAGACCTCGCCGCTCTTCTCCCACGGTACATGCTCCCCAATCGG ATCGTTGTTCTGGACCGCCTGCCACAGACTGCCAATGGTAAGATCGATCTGAAGGCTCTCCAGACCACCCAGGAA GCTCAGCTCACCGTGGGAGAGCGGGCTTTCATGGCCCCACGCACTCCCCTGGAACGTCGTATCCGTGACATCTGG CAGGCTGTCCTGAAGCGGGACCAGGTCTCCGTGACTGACGATTTCTTCGAACTCGGTGGAAACTCCCTGCTGGCC  GTGGCTCTGGTTTCGCGTCTCAACGCCGACTTCGGTGGAGCTATCCCCTTGCAGATCCTCTTCGAGGCTCCCACT GTGGAACGTCTCGCCGCCGCTCTTGAGGCTACTTCGCCGCGGCCTGCCTCCCGCCTGGTTCCACTGCAGCCAGAA GGCCGCGGTACTCCGCTCTACTGTTGGCCCGGTTTGGGCGGTTACCCCATGAACCTTCGGCCACTGGCTGCCGCC CTCGGTACTGAACGTCCTGTGCACGGTGTGCAGGCCCACGGCATCAACCCCGGCGAGTTCCCATACGATGATGTG CGTGCTATGGCCGCCGCCGATGTGGAAGCCATCCGGGAGATCCAGCCCCACGGACCGTACCTCCTGTGCGGATAC TCCTTCGGAGCTCGTGTCGCTTTCGAAGCTGCCCGGCAGCTGGAACAGGCTGGCGAGCAGGTCGAGCAGCTTTTC CTGGTTGCCCCAGGTCAGCCCCGTCTGCGGCCAGAGGACGCCGTGGGTGCTACTGGTCGGGCCGACTTCACTGAC CGGGCTTTCCTCGCTCTCCTTTTCTCGGTGTTCGCTGGAACTCTCAGCGGACCACGGCTCGACCAGTGTCTGCGG ACTGTTACTGACGAGGATGGATTCGTCGCTTTCGTGACCGCCTCGTTCCCAGGCCTGGGCGAGGAACTTGTTCGG GCCGTGACCGGTATCGTCCGGCGGACCTACTCGCTCACCTATGAGTTCCATGAACTCCGGGGACGGCGGCTGGAC GCCCCAGTTACCCTCGTGCGGGCTACTGACGACAACTATTCCTTCATCGAGCACGAGGGTGGATACTCCGCTCGG CCACCAGCCGTTCATCAGTTGCGCAGCGGACACTATGAGCTGCTCCGGGAACCCCATGTTGCCCGGCTTGCCGCT GTGCTTAATGATCGGCTCTCCGCTGGTCCCTCCACCTCGCCACGCCATTCCCAGCCCGCTCAGGCCACCGTCCAG GAAGTTGGAGTTCCACACATCAATATCAAGCACTTCCCCGTTTCTATCACTGAAGAAAAGGAACTGGAACTGGTG GCCGCCGTTACTACTGCTGTGCGCAACGCCTTCGGTTGCACTGAAGAGGTTGTTAGCATCGCCCTGGAGCCTGTG GCCCAGGAAGTTTGGAATGAGCGTGTCTACATCCCAGAGATTGTCGCCCGTCAAGAACTGCTCCGTAAAACGCCA  AACTACTGA |
