# Supplementary material for: Potential of Aspergillus oryzae as a biosynthetic platform for indigoidine, a non-ribosomal peptide pigment with antioxidant activity
Source: PLoS One. 2022 Jun 23;17(6):e0270359. doi: 10.1371/journal.pone.0270359 (PMC9223385; doi:10.1371/journal.pone.0270359)
Supplement: S2 Table — (DOCX) [file pone.0270359.s007.docx]

S2 Table. Overlapping primer sets used for amplifying specific DNA fragments by PCR.

| **Amplified fragment** | **Size**  **(kb)** | **Primer name** | **Sequence (5' to 3')** |
| --- | --- | --- | --- |
| AfpyrG | 2.3 | AfPyrG_F | TGAGAAGATGCGGCCAGCAAAACGAATTCGAGCTCGGTACCCGGCCGG |
|  |  | AfPyrG_R | CCGTTCTTCTTATTGATTTGGGCGCGCCTACTCTCCACAATCCACAC |
| PAngpdA | 2.3 | PAnGpdA_F | GTGTGGATTGTGGAGAGTAGGCGCGCCCAAATCAATAAGAAGAACGG |
|  |  | PAnGpdA_R | TACAAGGAATGGTTTCAACGCTCATGCTAAGCGGGCCGCGGGATGATGTC |
| AoinK | 4.3 | AoInK_F | ATGAGCGTTGAAACCATTCCTTGTAGCAGGCGTGCGGCTTTGGGAC |
|  |  | AoInK_R | TCAGTAGTTTGGCGTTTTACGGAGCAGTTCTTGACGGGCGAC |
| Tnos3 | 0.3 | TNos3_F | CTCCGTAAAACGCCAAACTACTGACCGGCTGCAGATCGTTCAAACATTTGG |
|  |  | TNos3_R | GCGCTTACACAGTACACGAGGACTGCGGCCGCAATTCTCATGTTTGACAGCTTATC |
